# Inflammatory and hematologic biomarker profiles across central branch and hemi-retinal vein occlusion

**DOI:** 10.6026/973206300212781

**Published:** 2025-08-31

**Authors:** Pankaj Kushwaha, Anjali Singh, Praher Shrivastava

**Affiliations:** 1 Department of Ophthalmology, Chhindwara Institute of Medical Sciences, Chhindwara, Madhya Pradesh, India; 2 Department of Ophthalmology, Atal Bihari Vajpayee Government Medical College, Vidisha, Madhya Pradesh, India

**Keywords:** Retinal vein occlusion, neutrophil-lymphocyte ratio, platelet-lymphocyte ratio, monocyte-HDL ratio, inflammation, ischaemia

## Abstract

Retinal vein occlusion (RVO) is a common vision-threatening vascular disorder, yet reliable biomarkers to predict its severity at first presentation are lacking. Therefore, it is of interest to assess complete blood counts and fasting lipids within 24 hours of diagnosis of 50 newly diagnosed Retinal vein occlusion (RVO) eyes and 50 controls. Neutrophil-to-lymphocyte ratios (NLR), platelet-to-lymphocyte ratio (PLR) and monocyte-to-HDL ratio (MHR) were calculated; severity was graded clinically and angiographically. Logistic regression adjusted for age, hypertension, diabetes and LDL and Receiver-operating characteristics (ROC) determined optimal cut-offs. Simple ratios derived from a haemogram strongly predicted RVO severity independent of traditional risk factors. NLR and PLR could be incorporated into first-visit triage to prioritize fluorescein angiography and early anti-VEGF therapy in high-risk eyes.

## Background:

Retinal vein occlusion (RVO) is the second most common sight-threatening retinal vascular disorder after diabetic retinopathy, with a global prevalence of 0.5-2 % [1]. Virchow's triad-venous stasis, endothelial dysfunction andhypercoagulability remains the conceptual bedrock for RVO pathogenesis [2]. Beyond classical risks such as hypertension, diabetes and glaucoma [3], mounting evidence emphasizes a systemic inflammatory milieu. Elevated C-reactive protein (CRP) and homocysteine have been repeatedly implicated, but their routine clinical use is limited by cost, turnaround time and variability [4, 5]. In recent years, composite blood-cell ratios obtainable from a standard complete blood count have emerged as inexpensive, reproducible inflammation markers in cardiovascular and cerebrovascular disease [6, 7- 8]. The neutrophil-to-lymphocyte ratio (NLR) integrates acute neutrophil-driven injury and relative lymphopenia; the platelet-to-lymphocyte ratio (PLR) couples thrombogenic platelet activity with immune status; and the monocyte-to-HDL ratio (MHR) reflects oxidative atherogenesis wherein monocytes and dysfunctional HDL interact [9, 10]. Ocular studies are fewer but suggest clinical relevance. Kumar *et al.* demonstrated higher NLR and PLR in RVO versus controls [11], while Lahiri *et al.* linked elevated MHR to branch RVO [12]. Yet most previous work was retrospective, pooled all RVO subtypes, or lacked systematic severity grading. Whether these ratios predict disease severity i.e., the ischaemic phenotype that portends macular oedema, neovascular complications and poorer visual prognosis remains unclear [13]. Therefore, it is of interest to quantify differences in NLR, PLR and MHR between RVO patients and matched controls and to evaluate the ability of these ratios to discriminate ischaemic from non-ischaemic RVO after adjusting for conventional systemic covariates.

## Materials and Methods:

## Study design and participants:

Observational case-control study conducted at the Retina Clinic, Department of Ophthalmology, S.S. Medical College, Rewa, India. Institutional Ethics Committee approval adhered to the Declaration of Helsinki; all participants gave written informed consent.

[1] Cases: consecutive adults (≥18 y) with first-episode RVO (central, branch, or hemi-retinal) confirmed by slit-lamp biomicroscopy and fundus fluorescein angiography within 7 days of symptom onset.

[2] Controls: age- (±5 y) and sex-matched cataract pre-operative patients without retinal vascular disease.

## Exclusion criteria:

Current infection, autoimmune disorders, haematologic disease, malignancy, chronic renal/hepatic failure, anticoagulant or antiplatelet therapy, steroids, pregnancy.

## Data collection:

Demographics, systemic comorbidities, blood pressure and intraocular pressure were recorded. Ischaemic RVO was defined by ≥10 disc-diameter capillary non-perfusion or afferent pupillary defect.

## Laboratory analysis:

Venous blood (8 a.m., fasting) was processed within 2 h.

[1] CBC-Sysmex XN-1000 analyser: neutrophils, lymphocytes, monocytes, platelets, mean platelet volume.

[2] Biochemistry-Olympus AU480: fasting lipids, creatinine, CRP, homocysteine.

[3] Indices:

NLR = neutrophils/lymphocytes

PLR = platelets/lymphocytes

MHR = monocytes (x10^3^/µL) / HDL (mg/dL)

## Statistical methods:

SPSS v24.0. Normality: Shapiro-Wilk. Continuous variables: mean ± SD or median (IQR); categorical: n (%). Group comparisons: t-test/Mann-Whitney, χ^2^. Multivariate logistic regression identified independent predictors of ischaemic RVO. ROC curves estimated the sensitivity/specificity. p<0.05 is significant.

## Results:

Cases and controls did not differ in age (57.1 ± 10.6 vs 60.1 ± 6.7 y, p=0.09) or sex (64 % male each). Hypertension (58 % vs 34 %, p=0.02) and diabetes (46 % vs 22 %, p=0.01) were more prevalent in cases. Median NLR, PLR and MHR values are summarised in Table 1 (see PDF). RVO eyes showed significantly higher NLR and PLR, whereas MHR differences were borderline. Twenty-three eyes (46 %) were classified as ischaemic. They exhibited greater CRP, homocysteine, NLR and PLR than non-ischaemic eyes (all p<0.01) but similar MHR (Table 2 - see PDF). Figure 1 (see PDF) depicts box plots; Figure 2 shows ROC curves.

[1] NLR ≥ 2.8: sensitivity 78 %, specificity 75 % for ischaemic RVO.

[2] PLR ≥ 150: sensitivity 70 %, specificity 80 %.

[3] Combined criterion (either high): sensitivity 91 %, specificity 69 %; AUROC 0.86.

After adjustment, NLR and PLR remained significant (Table 3 - see PDF). LDL, CRP and hypertension also contributed, but age, sex and MHR did not.

## Discussion:

In this case-control study we observed that patients with retinal vein occlusion (RVO) had significantly higher systemic inflammatory burden-reflected by elevated C-reactive protein (CRP) and plasma homocysteine-together with an atherogenic lipid milieu (higher total cholesterol, triglycerides, LDL, VLDL and lipid ratios; lower HDL) compared with age- and sex-matched controls, while composite blood-cell indices showed a mixed pattern: platelet-to-lymphocyte ratio (PLR) and mean platelet volume (MPV) were increased, neutrophil-to-lymphocyte ratio (NLR) was not significantly different overall and monocyte-to-HDL ratio (MHR) did not differ globally but trended higher in branch disease. These findings reinforce the multifactorial model in which venous stasis, endothelial dysfunction and hypercoagulability interact with systemic vasculometabolic insults to precipitate retinal venous thrombosis [1, 2]. Our CRP signal accords with prior reports linking acute-phase activation-and, in some series, CRP/albumin ratio-to RVO, particularly in hypertensive subjects, underscoring inflammatory-haemorheologic crosstalk [6]. The robust homocysteine elevation we detected across RVO subtypes and especially in the small HRVO subset, mirrors cumulative evidence that hyperhomocysteinaemia promotes endothelial injury and augments thrombotic risk in retinal vascular occlusive disease; several groups have shown stronger or more frequent abnormalities in central events, though data are heterogeneous [8, 11]. Although our overall MHR signal was null, prior BRVO cohorts have reported higher MHR, suggesting possible subtype specificity or threshold effects not captured in our sample [12, 13]. We found PLR-integrating platelet activation with relative lymphopenia-to be markedly higher in cases, aligning with multi-centre datasets that implicate both PLR and NLR as low-cost inflammatory markers in RVO, even if our NLR difference did not reach significance, perhaps due to variance and modest size [14, 15-16]. MPV was significantly elevated, supporting platelet reactivity in pathogenesis; literature shows predominantly positive associations, though one study reported lower MPV, highlighting assay and timing influences [17, 18]. The strong dyslipidaemic signature we observed parallels classic and contemporary work linking abnormal lipoproteins to retinal venous occlusion and supports routine metabolic screening in these patients [19, 20]. Limitations include small, hospital-based sampling (notably few HRVO), single time-point, non-fasting draws and unmeasured confounders (e.g., hypertension control, renal status) that could attenuate or inflate associations; larger, longitudinal studies with multivariable adjustment are warranted.

## Conclusion:

Elevated NLR and PLR emerged as robust, independent predictors of ischaemic retinal vein occlusion. These low-cost indices, derivable from a routine haemogram, outperform traditional lipid ratios and when used together provide excellent discrimination (AUROC 0.86). Incorporating NLR and PLR into initial RVO work-ups could expedite risk-stratified management, especially where angiography resources are limited. Future multicentre validation and longitudinal tracking will solidify their role in clinical algorithms.
